# Supplementary material for: Are carbon emissions trading and green financial instruments synergistic? -Comprehensive quantitative research based on content analysis
Source: PLoS One. 2024 Mar 7;19(3):e0298601. doi: 10.1371/journal.pone.0298601 (PMC10919601; doi:10.1371/journal.pone.0298601)
Supplement: S1 Data — (ZIP) [file pone.0298601.s001.zip › Data/地级市Z_F工作报告.zip!/┴╔─■/┤≤┴1⁄4╩╨/└┤╫╘╣1⁄2╓┌║┼í╛┬φ┐╦╩2╛▌═°í┐_2006.docx]

各位代表：

　　我代表市政府向大会报告工作，请予审议，并请市政协各位委员提出意见。

　　2005年和“十五”时期工作回顾

　　2005年是我市加快老工业基地振兴和“大大连”建设的关键之年。在党中央、国务院，省委、省政府和市委的正确领导下，市政府坚持以科学发展观统领全局，带领全市人民抢抓机遇，加快发展，国民经济和社会事业取得了新成就，市十三届人大三次会议确定的各项任务已全面完成。

　　预计全市实现地区生产总值2290亿元，比上年增长14.2％；完成全社会固定资产投资1110亿元，增长55％；完成地方财政一般预算收入151.4亿元，可比增长18.7％；实现社会消费品零售总额732亿元，增长13.5％；完成自营出口124.3亿美元，增长22.4％；实际利用外资30亿美元，增长36.4％；城市居民人均可支配收入11934元，农村居民人均纯收入5900元，分别增长15％和15.6％。

　　2005年，我们重点做了以下工作。

　　一、加大投入，全力推进，国际航运中心建设全面提速。按照《大连东北亚国际航运中心建设规划》，加大投入，重大基础设施项目的投资比上年增长66.8％。50个港口重大基础设施建设步伐加快，大窑湾集装箱码头二期工程2个泊位投产、2个泊位主体工程完工，三期工程开工建设；“一岛三湾”核心港区海上养殖清理工作全面展开；大窑湾北岸围堰提前合龙；大连湾、大窑湾航道改扩建和大窑湾港区北防波堤工程顺利推进；大连湾通用杂货泊位、保税区汽车码头等项目进度加快。区港联动试点稳步推进，保税港申办工作开始启动；沈阳、通辽、满洲里内陆干港建设进程加快，新开通了2条集装箱班列。大孤山国际物流园已基本具备国际中转、配送、采购和转口贸易功能。航运交易市场服务功能不断完善。大连港东部港区搬迁全面启动。大连港口完成货物吞吐量1.7亿吨，增长17.6％；集装箱吞吐量268.6万标箱，增长21.5％。完成了长兴岛开发总体规划和三个专项规划编制，公共港区建设全面开工，基础设施配套建设快速推进。烟大火车轮渡铁路、码头主体工程竣工，土羊高速公路、大窑湾疏港高速公路、沈大与丹大高速公路连接线和金窑铁路复线开工建设。周水子机场军转民工作取得突破，机场二期扩建工程完工，与南方、东方航空公司签署了战略合作框架协议，新开通国际航线12条，空港旅客吞吐量达到541万人次，增长17.2％。数字口岸建设步伐加快，港检、关检联动合作进一步加强，“大通关”建设取得新进展。

　　二、突出重点，扶优做强，现代产业基地初具规模。推进产业布局和结构的战略性调整，加大工业投资力度，“四个基地”、八大产业集群和一批重点工业项目建设加快，工业经济后劲增强。重工起重铸钢扩容改造等373个项目竣工，全年完成工业投资367亿元，比上年增长89％。大连石化和西太平洋石化扩能改造已基本形成3000万吨炼油能力。国家战略储备油基地部分储罐开始安装。旅顺南路软件产业带进展顺利，国家级动漫游产业基地、光产业园55万LED芯片用外延片等项目开工建设。又有8家世界500强软件企业落户大连。丰田工机创新汽车部件、德国FEV发动机研发中心等项目建成投产，一汽大众30万台轿车发动机、大柴道依茨汽车发动机等项目开工建设。承担了大型隧道掘进机等12项国家重点工程的装备研制，装备制造业整体水平有新提高。造船重工和新船重工整合重组的大连船舶重工集团公司，成为中国造船工业的“旗舰”。中远30万吨修船坞基本完工。十大工业园区完成基础设施投资30亿元，引进外资项目40个，内资项目112个，产业集群发展态势良好。大钢、大化搬迁改造工作全面启动。全市规模以上工业完成总产值2491亿元，增长23％，增加值增长21.2％。实现高新技术产业产值1320亿元，增长30.7％；软件销售收入100亿元，出口3亿美元，分别增长43％和50％。“四个基地”产业带动作用强劲，完成总产值占全市规模以上工业产值比重达74.5％，增长23.4％。市属国有大中型企业股份制改革有突破性进展，基本建立起国有资产监督管理体系。大连产权交易所成为省级交易中心。

　　星海湾金融商务区建设全面推进，期货大厦建设进度加快。特色便民商业建设和“千村百镇”市场工程全面展开，城乡流通现代化体系不断完善，我市成为全国“三绿工程”样板城市。全年接待海外游客60万人次，旅游外汇收入4亿美元，旅游总收入210亿元。举办各类展会123个，成交额323亿元。

三、抢抓机遇，强化招商，对外开放再上新台阶。我市充分利用国办36号文件的优惠政策，强化招商引资工作。承办了“WTO小型部长会议”等20多个大型国际会议，举办了“韩国重点工业园区招商说明会”等30多个大型经贸活动，接待了70多个国家和地区重要经贸团组400多个，客商10多万人次，引进外商投资企业1058家，其中1000万美元以上的项目235家，跨国公司投资明显增加，德国大众等17家世界500强企业在连新办了19家企业。新引进跨国公司地区总部、研发中心、投资性公司24家。服务业利用外资取得新突破，新批服务业外资项目421个，合同外资18.5亿美元。先导区带动作用增强，新批外商企业数、合同外资数、实际利用外资额分别占全市的47.9％、59.5％和58.1％，引进大项目占全市的72.3％。出口结构不断优化，机电产品、高新技术出口分别占出口总值的44％和24％，有4个出口品牌被评为国家名牌。对外经贸合作步伐加快，全年签订对外承包和劳务合作合同820项，合同金额3亿美元；新成立境外企业19家、办事处4家。

　　四、积极扶持，优化结构，县域经济快速发展。加大对县域经济的支持力度，向“三农”投入16.6亿元，农村固定资产投资增长86.7％。农林牧渔及服务业实现总产值340亿元，增长15％。农业结构进一步优化，五大优势产业所占比重提高到90％，农产品质量和效益明显提高，我市农产品质量安全总体水平居全国前列。采取有力措施，有效抵御了高致病性禽流感传播。农业产业化经营规模扩大，新发展农产品加工龙头企业34家，新增省级重点龙头企业10家。农村造林绿化生态建设全面推进，新增加林木1亿株。渔业继续保持快速增长。积极扶持县区工业园区发展，安排软贷款26.7亿元，搭建了11个政府投融资平台、3个中小企业平台，有效带动了社会资金的投入，农村工业化程度、发展后劲大幅提升，县域经济出现了多年未见、多项指标大幅增长的好形势，有36个乡镇（街道办事处）跻身全省百强乡镇行列。全年新办民营企业1万多户，引进内资150亿元；民营经济营业收入、增加值、实缴税金分别增长23％、21.5％和28.1％，增加值占全市GDP的比重达51.2％。

　　五、加快建设，改善环境，城乡建设迈出新步伐。按照《大连市城市总体规划》加大城市基础设施建设力度，城市综合功能全面提升。全市安排城建重点项目162项，完成109项，实现投资额200亿元。泰山热电厂建成并投入运营，世界博览广场、市游泳馆竣工投入使用，庄河电厂开工建设，红沿河核电站实施“五通一平”建设。“西拓北进”战略成效明显，甘井子区城市化进程加快，旅顺口区、金州区基础设施建设力度加大。城八线瓦房店段、海皮路三期、大窑湾海鲇路、盖亮线改扩建、鹤大线大修及黑大线样板路工程竣工；城区西部通道、椒金山隧道改扩建工程、快轨金州线全面开工建设；完成了解放路等10条干路的拓宽改造，胜利桥等三座跨铁路桥开工建设；完成村通油路1080.4公里，又有218个行政村通油路。生态环境建设及保护力度加大，启动了城市规划环境影响评价工作，老虎滩和泉水2座污水处理厂建成，凌水河等4座污水处理厂开工建设。凌水河区域环境综合整治基本完成，大化棉花岛渣场治理工程稳步推进。继续加大烟尘综合整治力度，拆除锅炉203台、烟囱165根，并网供热面积增加202万平方米。新建星级公共厕所150座。城区新增绿地90万平方米，城市绿化覆盖率达到42.3％。我市连续第五年荣获全国畅通工程评比第一名。

　　六、注重民生，促进和谐，人民生活不断改善。坚持以人为本，从群众最关心的现实问题做起，使老百姓得到了更多的实惠。实现城镇就业16万人，城镇登记失业率控制在4％以下。全年发放下岗失业人员小额担保贷款1.8亿元，有5000余人实现了自主创业；2361户无就业家庭达到了每家至少有1人就业。全年发放城乡居民最低生活保障金1.59亿元，救助城乡困难居民127万人次。全市募集慈善资金9630万元，实施援助项目20余个。出台了《大连市农村居民最低生活保障办法》。108万农业人口参加了新型农村合作医疗，农村低保对象全部纳入新型农村合作医疗。在国内率先将1.3万名享受国家抚恤补助的在乡重点优抚对象纳入城镇职工医疗保险，我市医疗救助经验在全国推广。加大旧城区改造力度，拆迁改造了石门山、春华山等9个旧居民区，为4.35万户市民改善了居住条件。开工建设54.9万平方米经济适用住房；以廉租方式解决了840户“双特困”家庭住房问题；解决残疾人困难住房309户；为1.8万户家庭办理住房公积金贷款22亿元。完成街巷路改造804条、151万平方米。甘井子区50条摸黑路全部安装上路灯。市区改造、新建煤气管网43.2公里，解决居民住宅煤气配套3万余户。新增供热面积396万平方米。接收管理二次加压泵站318处，完成居民室内供水管网改造和水表出户5万户。投资2200万元购置机械化环卫清扫设备，城区道路机扫率由13％提高到40.3％。新建农村饮水工程179项，解决了20.3万人饮水困难。设立5000万元安全生产专项资金，建立起食品、药品、特种设备等安全生产监控体系。

　　七、统筹兼顾，协调发展，各项社会事业全面进步。过去的一年，各级政府更加注重经济与社会事业的协调发展。全年实施科技计划项目462项，申请专利5573件，获国家和省科技进步奖54项。首次设立了最高科学技术奖和科学技术功勋奖。清华光洋数控技术工程化联合实验室成立。我市成为全国首家软件版权保护示范城市。城乡办学条件和教育水平有新提高，高中阶段新生入学率达到91.5％。设立1000万元专项资金推进城区义务教育均衡发展、拨款2300万元专项补助农村中小学公用经费。投入8000万元，初步建成了覆盖全市的远程教育网络，中小学信息化工程取得重要进展。盲聋学校和残疾人职业技能培训中心建成并投入使用，旅顺大学园配套工程基本完成，辽师大新校区、沈阳音乐学院和鲁迅美术学院大连校区开工建设。深入实施人才强市战略，引进海内外人才1.6万人，完成国家引智项目94个，培训各类专业技术人员8万人次。文化事业进一步繁荣，大连艺术学校获得3个国际大奖，庄河市被评为全国文化先进地区。广播电视人口覆盖率达到99％。完成有线电视数字化整体转换22万户。启动公共卫生体系三年规划建设，市财力投入2.7亿元，新建和改造了67所乡镇卫生院、5所急救分中心、27个急救分站、6所妇幼保健院，农村医疗卫生基础设施显著改善；新传染病医院、药品检验所大楼主体工程完工。继续保持低生育水平，计划生育率为99.46％，人口自然增长率为－0.02‰。体育事业蓬勃发展，成功举办了第三届国际徒步大会、第十九届国际马拉松赛和第十届大连市运动会，大连实德队夺得中超联赛和足协杯两项冠军，实德女足夺得全国锦标赛冠军。新闻出版、民族、宗教、人防、侨务、对台、妇女儿童、残疾人、老龄、民兵和预备役等工作均取得喜人成绩。

　　八、以创促建，依法管理，精神文明、民主法制和服务型政府建设成效显著。全面开展文明城市创建活动，经过全市人民的共同努力，我市荣获首批“全国文明城市”称号。双拥共建工作取得新成绩，我市军嫂进社区工作经验在全国推广。各级政府自觉接受人大、政协和人民群众的监督，充分发挥各民主党派、工商联、无党派人士参政议政作用，1361件市人大代表建议和政协提案办理完毕。市政府与市总工会联席会议制度正式启动，积极支持共青团、妇联等群团组织的工作。全面落实行政执法责任制和执法过错追究制，加大“四五”普法和依法治市工作力度，出台了《大连市法律援助办法》，民主法治示范村、示范社区创建活动成效明显。强化监察、审计监督，全年处分违反政纪公务员256人。继续严厉打击各种犯罪活动，加强社会治安综合治理，妥善处理各种矛盾，全市政治安定、社会稳定。完成了《大连市国民经济和社会发展第十一个五年规划纲要（草案）》和11个专项规划编制工作。继续削减和调整行政许可项目，调整职能交叉，政府部门职能进一步规范，服务水平和行政效能不断提高。建立了新闻发布制度，政务公开工作得到加强。推广全程办事代理制、网上审批等经验，全市“一站式”服务效率和水平进一步提高。认真办理群众来信来访，全市信访处结率达95％。

　　各位代表，2005年是“十五”计划的最后一年。五年来，我市坚定不移地把发展作为第一要务，聚精会神搞建设，一心一意谋发展，克服了国际经济低迷、能源紧缺、“非典”等不利因素的影响，保持了经济与社会全面协调、可持续发展，城市功能全面优化，人民生活水平不断提高，国内外影响日益扩大，“十五”计划的各项目标和任务圆满完成。

　　五年间，全市累计实现地区生产总值8526亿元，年均增长14.3％。完成地方财政一般预算收入573亿元，全社会固定资产投资3000多亿元，社会消费品零售总额2908亿元，分别比“九五”时期增长103.6％、140％和68.5％。完成外贸自营出口440亿美元，比“九五”时期增长87.8％；实际利用外资107.8亿美元，占改革开放以来总和的53.9％。港口吞吐量跨入亿吨大港行列。规模以上工业增加值比“九五”时期增长1.3倍。非公有制经济增加值超过全市经济总量的一半，为区市县创造了80％的财政收入。城乡建设取得重大成就，完成了引英入连供水工程、30万吨级原油码头、30万吨级矿石码头、火车站和机场改扩建、城市快轨交通等一批重大基础设施建设项目，城市功能明显提升，综合经济实力显著增强。科技、教育、文化、卫生、体育等各项社会事业全面进步。人民生活不断改善，“十五”期末，城市居民人均可支配收入和农民人均纯收入分别是“九五”期末的1.7倍和1.6倍。

　　各位代表，过去的五年，大连发生了令人鼓舞的巨大变化，这是省委、省政府和市委正确领导的结果，是全市人民团结奋斗、开拓进取的结果，也得益于历届市委、市政府打下的良好工作基础。在此，我代表市政府，向在各个领域和岗位上无私奉献、辛勤工作的同志们表示崇高的敬意！向给予政府工作大力支持的人大代表和政协委员及各民主党派、工商联和无党派人士，向关心和支持政府工作的离退休老同志，向中省直各部门和兄弟省市驻连机构，向驻连部队和武警官兵，向所有关心和支持大连建设发展的海内外朋友们，表示衷心的感谢！

　　各位代表，我们有理由为过去五年的成就而感到自豪，同时我们也清醒地认识到大连正处在新的发展时期。我们必须在世界经济、东部沿海经济和东北振兴的大格局中审视大连的发展与进步，直面发展中所存在的困难与问题。一是经济运行质量不够高，经济增长方式粗放、经济结构不尽合理的情况还没有得到根本性的改善。二是企业自主创新的动力、能力不足，拥有自主知识产权和知名品牌的产品较少。三是环境和资源对我市经济社会快速发展的制约增加，建设资源节约和环境友好型社会的任务繁重。四是城镇就业和再就业压力仍然较大，收入分配不尽合理，城乡公共事业发展不够均衡问题仍然突出。五是投资软环境还需进一步改善，一些政府部门公共服务和社会管理的能力还不够强。面对这些问题，我们有信心加以克服与解决，而且我们将立足于用发展的方法破解这些矛盾与困难。

　　“十一五”发展目标和2006年政府重点工作任务

　　各位代表，未来五年将是经济快速增长和社会结构加速转型的重要历史时期。我们既面临着难得的发展机遇，又必须应对严峻的挑战。经济全球化的加速，国内经济持续发展，老工业基地振兴战略的实施，将全面带动大连较快发展；对外开放的进一步扩大和东北亚重要国际航运中心建设的全面推进，将为大连广泛参与国际合作与竞争提供更多商机；改革的不断深化和社会主义市场经济体制的逐步完善，将为大连加快发展提供强有力的体制保障；人民生活水平的不断提高，消费需求快速增长和消费结构拓展升级，将为优化产业结构创造巨大的发展空间；和谐社会的构建，精神文明、政治文明和民主法制建设的推动，将进一步激发全市人民的创造热情和活力。同时，随着经济社会的快速发展，资源和环境制约加大、总供给与总需求不平衡、微观经济低效运行等体制性、机制性、结构性的矛盾和问题将进一步凸现，我们必须有足够的认识，增强历史责任感和紧迫感，树立强烈的忧患意识，把握机遇，迎接挑战，加快发展。“十一五”时期我市总的发展思路是：坚持以邓小平理论和“三个代表”重要思想为指导，用科学发展观统领全局，紧紧抓住振兴东北老工业基地和进一步扩大对外开放的双重机遇，以建设东北亚国际航运中心、新型产业基地和社会主义新农村为重点，深化改革，扩大开放，加快经济结构调整，转变经济增长方式，全面建设和谐社会，提高全市的综合经济实力、自主创新能力和国际竞争能力。主要发展目标是：五年间，地区生产总值年均递增13％左右，万元地区生产总值综合能耗和水耗下降20％以上；地方财政一般预算收入年均递增13％左右；全社会固定资产投资年均递增16％左右；社会消费品零售总额年均增长13％左右；外贸自营出口总额年均递增15％左右。努力使城乡居民收入水平与GDP保持同步增长。到2010年，港口年吞吐量达到2.5亿吨左右，集装箱年吞吐量达到800万标箱，迈入国内沿海港口五强行列。城镇登记失业率控制在4％以内。年人口出生率和人口自然增长率分别控制在6.8‰和0.64‰以内。到“十一五”期末，率先实现老工业基地全面振兴，率先基本实现建设社会主义新农村的要求，率先基本实现社会主义现代化。市政府制定并经市委九届十一次全会讨论的《大连市国民经济和社会发展第十一个五年规划纲要（草案）》将提请会议审议。

　　2006年是“十一五”规划的开局之年，政府工作的总体要求是：认真贯彻党的十六大、十六届三中、四中、五中全会和中央及省经济工作会议精神，深化改革，扩大开放，推动创新型城市建设；加快经济结构调整和经济增长方式转变，努力提高经济增长质量和效益；强化大项目牵动，加快“一个中心、四个基地”建设进程；坚持区域协调发展，扎实推进社会主义新农村建设。主要预期目标是：地区生产总值增长13％，力争14％；万元地区生产总值综合能耗和水耗降低4％，力争5％；地方财政一般预算收入增长13％，力争14％；固定资产投资增长20％，力争30％；社会消费品零售额增长13％左右；外贸自营出口增长15％左右；实际利用外资增长30％左右；城市居民人均可支配收入和农民人均纯收入分别增长13％左右；城镇登记失业率控制在4％以内。

　　为完成上述目标，要重点抓好以下工作：

　　一、加快国际航运中心建设，全面提升城市服务功能。加快航运中心核心功能区的建设。要完成保税区汽车码头、大连湾通用杂货码头和大窑湾二期4个泊位、三期2个泊位工程；全力推进第二个30万吨油码头和矿石码头二期建设；加快大窑湾北岸开发进度，完成北部海域清理和北岸动迁工作；大连湾航道、大窑湾北防波堤完成主体工程。加快长兴岛开发建设，完成长兴岛大桥、公共港区水电配套和3个码头水工建设；快速推进大型深水专业化码头和入岛铁路一期工程。完成大连港东部港区搬迁工作。加快推进大窑湾疏港路和土羊高速公路建设。烟大铁路轮渡工程竣工投产；启动哈大客运专线动迁工作；推动东部铁路及庄岫铁路前期工作；金窑复线达到年度建设目标。加快空港建设，新开通国际航空航线5条，增加直达日、韩航线和北京、上海等国内大城市的航班密度。大连港要本着互利共赢的原则，加强与周边港口的合作，努力形成共同打造国际航运中心的整体格局。加强区港合作，拓展区港联动试点，推进保税物流业务与口岸物流业务的整合，将大窑湾南岸集装箱码头、汽车码头及后方区域纳入试点范围，把保税政策和功能扩展到油品、粮食、矿石等专业港区。建立保税物流网络和运作机制，推动与东北腹地内陆干港联动，不断提升大连口岸的辐射力。争取大连保税港区申办成功。

　　大力发展现代服务业，构建完善配套的城市综合服务功能和国际航运中心支撑体系。加快推进星海湾金融商务区建设，期货大厦年内主体封顶，启动后续工程项目。积极引进各类中外金融机构和跨国公司管理总部，落实好花旗银行、渣打银行和荷兰银行的引进工作。充分利用保税物流园区、北方粮食物流中心、大连陆港物流基地、香炉礁物流园区等物流网络，开拓腹地资源，建设好物流分拨配送中心。进一步推动商贸流通业的现代化和国际化水平，重点吸引跨国公司入驻大连设立地区总部、跨国商品采购中心、物流配送中心。继续推进以连锁经营、物流配送、特许经营和电子商务为代表的现代流通方式，进一步完善社区商业服务网络。充分发挥星海会展中心、世界博览广场以及金石滩旅游度假区的服务功能，促进会展和旅游业发展。举办好中国国际服装纺织品博览会、进出口商品交易会等大型国际性展会。积极拓展国内外旅游市场，创新旅游产品，全年接待海外游客增长14％，实现创汇增长14％，旅游总收入增长16％。

　　二、培育自主创新能力，切实转变经济增长方式。全面贯彻《国家中长期科技发展规划纲要》，大力推进自主创新，建设创新型城市。有计划按比例逐年增加科技研发投入，积极引导和鼓励企业增加研发经费，构建多元化公共科技支撑平台，成立10家工程技术研究中心、重点实验室和企业研发中心，科技创业、风险投资总规模均比上年增长50％。加快高新园区技术创新平台建设。充分发挥科研院所和大专院校的研发、人才优势和企业的生产优势，建设中科院大连科技创新园，新建5家二次科技孵化产业基地。加强知识产权保护，专利申请量突破6500件。

　　扶持“四个基地”的优势骨干企业，着力抓好一批产业关联度高、带动能力强的大项目。努力推动石化下游产业发展，继续推动百万吨乙烯项目前期工作。要加快推进国家半导体照明工程产业基地、旅顺南路软件产业带和动漫游产业基地建设，培育50项具有自主知识产权的软件产品。要集中力量组织好船用曲轴、风电设备、数控机床及功能部件等10个国家重大技术装备项目的开发攻关，加快我市由装备制造基地向先进装备研制基地转变。要继续扩大造船能力和配套能力，重点推进船用柴油机、船用推进器的扩能改造。全力推进中远造船落户大连。同时，鼓励和支持服装、生物制药、家具、新型建材、农产品深加工等一批市场潜力大、有竞争优势的消费类产品的发展。

　　大力发展循环经济。做好开发区循环经济试点和七贤岭产业化基地升级改造工作，推进一批循环经济重点项目，树立一批示范工程。加强重点耗能大户的节能改造和考核管理，降低工业万元产值综合能耗，资源节约和综合利用创效益3亿元。搞好海水淡化、中水回用、建筑节能等新技术、新工艺推广利用和太阳能、风力发电、水源热泵等节能产品的开发应用，建立产品能源标准指标体系，发展节能产品品牌。用循环经济理念指导工业调整改造，促进经济增长方式由粗放式向集约式转变，提高经济运行质量和企业经济效益。

　　三、扎实推进社会主义新农村建设，促进县域经济全面发展。要把提高农村生产力水平和增加农民收入作为重中之重，继续实行各项支农惠农的政策措施，加大向农村的投入，实现农村固定资产投资增长20％，农林牧渔及服务业总产值增长15％。

　　按照“生产发展、生活宽裕、乡风文明、村容整洁、管理民主”的要求，推进社会主义新农村建设。制定我市建设社会主义新农村实施纲要，选择一批乡村先行试点。加强对试点工作的指导，确定标准，规范建设，逐步推广。加大对农村科技、教育、文化、卫生事业投入，促进农村各项社会事业发展。

　　加强农业设施建设，调整农村经济结构。发展节水型农业灌溉面积6万亩。新发展规模化畜牧业饲养小区100个，果园面积20万亩，保护地8万亩。建设6个现代化花卉园区和10个规模化出口基地。继续实施渔业布局和结构调整6个“百万工程”，加强渔港和渔业支撑体系建设。全面启动五大林业工程，建设庄河银石滩国家级森林公园和瓦房店市龙门汤、甘井子大黑石省级森林公园。

　　加快推进农业产业化经营。进一步做强做大龙头企业。大力发展各类农民合作经济组织、中介服务组织和农产品出口经纪人队伍。实施农业标准化生产，开展农产品质量安全例行监测和信息发布工作，强力推进农产品认证认可工作，完善农产品市场准入制度。深入实施科技兴农计划，完善农业科技推广体系，继续实施十大增产技术，加大农业新品种、新技术的引进推广力度。

　　加快县域经济发展，提高自主增长能力。结合工业布局调整，继续引导大项目向区市县工业园区转移，加快农村工业化进程。继续推进北三市开发。加强农村供水、供电、交通等基础设施建设的扶持力度，抓好城乡对口帮扶工作，鼓励和引导城市科技、教育、卫生等人才到农村工作，支持小城市和重点镇的发展，逐步建立起乡镇垃圾和污水处理管理体系，加快农村城市化进程。认真做好我市第二次全国农业普查工作。

　　四、按照生态宜居城市的标准，提高城市建设与管理水平。充分发挥大连城市建设的良好基础和品牌优势，按照生态环境良好、配套设施完善、商务环境优越、城市管理规范的目标，加快生态宜居型城市建设。全市安排城建重点项目201项，计划完成投资257.9亿元。加快实施“西拓北进”战略，重点推进主城区向甘井子区拓展，搞好水、电、气和路网等城市基础设施建设，完善和提升主城区功能。进一步完善交通基础设施，完成西部通道、明珠路一期、朱棋路、新椒金山隧道、3座跨铁路桥改扩建等路桥工程；加快快轨金州线建设进度；拓宽改造西北路等10条中心城区交通干路；新开3条公交线路，启动201路有轨电车改扩建工程；启动新老市区第二通道项目前期工作。开工建设沿海公路。完成村通油路800公里，全面实现村村通油路。

　　抓好中心城区建设与管理。按照建设国际旅游港和商务中心的思路，全力推进大连港东部港区的开发建设；加快南北复兴里、石门山、春华山旧区拆迁后的规划建设与综合开发；除计划中的旧区改造外，坚持老城区“零开发”原则，保护好大连特有的城市风貌和建筑历史文脉。

　　高度重视生态环境建设与保护。全面推进甘井子工业区环境综合整治，加快大钢、大化整体搬迁进度，关闭31个矿山，高标准做好关闭矿山区域的规划和复垦。对砬夏河、梭鱼湾、河口湾、凌水湾区域、朱棋路两侧、棉花岛渣场及周边工业区环境进行综合整治。建成凌水河污水处理厂，加快马栏河污水处理厂二期、春柳河污水处理厂二期、夏家河污水处理厂及各污水处理厂的配套工程，市区生活污水集中处理率提高10个百分点；开工建设城市生活垃圾焚烧厂。加大烟尘区域综合整治力度，继续做好拆炉并网工作。实施污染企业排污许可证制度。开工建设梭鱼湾公园、西山和西郊森林公园。提高城市绿化美化水平，城区植树50万株，新增公共绿地150万平方米，绿化覆盖率达到42.8％，人均公共绿地面积增加到10.6平方米。

　　开工建设泰山热电厂二期、香海热电厂二期、甘井子热电厂等工程。完成大沙沟净水厂改造工作、启动三道沟净水厂一期改造工程；实施旅顺南、北路及周水子机场前供水工程建设；加快大伙房水库引水工程前期工作；完成水网改造73公里任务，改造居民室内水管5万户，全面完成二次加压泵改造。开工建设煤气新厂二期工程，完成煤气网管新铺和改造40公里。

　　加大城市管理工作力度。综合治理好占道经营、广告牌匾乱设、机动车乱停乱放、户外乱堆乱挂等问题。确保我市在国家畅通工程评比中继续保持领先地位。

　　五、进一步扩大利用外资规模，全面提高对外开放水平。要以世界的眼光，推动我市经济全面对外开放。充分发挥先导区在对外开放中的带动作用。开发区要进一步拓展发展空间，着力引进国际大公司重大制造业项目，力争在芯片制造、汽车及零部件上有所突破；高新技术园区要以创新体系建设为重点，积极培育研发能力，重点引进国内外研发机构、信息和生物技术、软件产业和信息服务业等高新技术产业；保税区要重点引进现代物流业和港口运输、国际贸易及临港加工业，加快建设国际物流园；各区市县要充分利用业已启动的工业园区，按照全市的产业规划，全面展开招商引资，迎接国际、国内的产业转移，带动外向型经济发展；市内各区要全力推进现代服务业招商引资，大力发展楼宇经济和总部经济。结合新一轮城市建设，加大房地产业利用外资力度，提高城市建设的国际化水平。要进一步提高利用外资质量，引导外资参与国企改革、城市基础设施和公用设施建设。吸引更多跨国公司在我市建立研发中心、技术服务中心、物流采购中心和地区总部。

　　进一步优化出口产品结构，扩大具有自主知识产权、自主品牌商品的出口，提高机电产品和高新技术产品出口比重，积极促进大型成套设备、船舶、石化和通信电子等产品出口，不断提高出口商品和服务的技术含量及附加值。进一步加强国际经贸合作，扶持一批骨干优势企业“走出去”发展。积极应对国外技术贸易壁垒，按照国际通用法则，加快建立技术贸易性措施体系。

　　六、深入推进各项改革，在重点领域和关键环节取得新突破。继续推进行政管理体制改革，加快政府职能转变，整合优化部门内设机构，切实把政府的管理职能转变到为市场主体服务和创造良好发展环境上来。健全和完善公共财政体制，搞好部门预算、政府收支分类和国库集中收付等财政制度改革，逐步建立财政绩效评价体系。推进商业银行跨区域经营，完成农村合作银行组建工作，探索商业保险参与社会管理的有效形式。

　　加快投融资体制改革，出台《大连市企业投资项目核准办法》等配套文件，规范政府投资决策规划和程序，建立政府投资项目招标核准、投资责任追究和社会监督等制度。推行非经营性政府投资项目代建制试点工作。加强投融资平台建设，整合改造政府系统投资公司，增强资本运作能力，为全市重要产业和公用基础设施发展筹集更多资金。加快机场、铁路等基础设施建设的社会化融资，实现投资主体多元化。

　　继续推进市属国有大型企业股份制改革。全面完成工商大型企业改革任务，公用事业单位改革要取得重大突破。促进国有大型企业与国际大公司、国内优势企业结为战略合作伙伴，形成多元化投资主体。进一步规范完善公司法人治理结构，健全外派董事和派出监事会制度。全力推进大连港股份有限公司等企业上市工作，做好股权分置改革。进一步完善国有资产监督管理体系，加快构建国有资产营运机构。

　　积极稳妥推进事业单位改革。启动行政管理类事业单位改革试点工作。深化公共卫生体系改革，全面推进新型农村合作医疗制度，逐步扩大农村社区卫生服务试点。积极推进文化体制改革，逐步将新闻媒体中的广告、印刷、影视制作等部分从事业体制中剥离出去。

　　鼓励民营企业参与国企改革，放宽市场准入条件，为民营企业创造更大的发展空间。政府各部门要转变作风，提高工作效率，为民营企业提供良好服务，解决好民营企业办事难、融资难等问题。进一步优化鼓励和支持民营经济发展的法制、政策和体制环境。继续实施发展民营经济“三个一”工程。民营企业增加值、营业收入、上缴税金等指标增长15％以上，固定资产投资、实际利用外资增长20％。新成立中小企业1万户，新增规模以上工业企业200户。

　　七、努力为市民办实事，不断提高人民群众生活水平。继续把事关人民群众生活的重要问题列入今年为民办实事工程，认真解决好改革发展过程中人民群众切身利益问题。进一步采取积极有效措施，努力提高城乡居民的收入水平。实施积极的就业政策，千方百计扩大就业和再就业，实现就业再就业14万人。大力实施职业素质提升工程，开展普惠制培训，提高就业质量。积极稳妥推行劳动合同制度和集体协商、集体合同制度，建立多层次的劳动争议协调体系。进一步完善社会保障制度，扩大基本养老保险覆盖范围，逐步建立失地失海农民社会保障制度。

　　努力改善城乡居民生活条件。建设经济适用房50万平方米。解决1000户“双特困”家庭廉租住房问题。帮助农村特困群众和贫困残疾人改造危房1500户。健全完善社会救助、社会福利和慈善事业相衔接的社会救助体系。进一步完善城市困难居民基本医疗救助制度。加快社会福利事业社会化进程，启动市老年福利产业园区项目建设。建设农村区域性中心敬老院25所。新建农村饮水工程100项。

　　深入整顿和规范市场秩序。强化对食品、药品监管，完善食品安全保障体系，继续深入推进“三绿”工程、食品放心工程和农村药品供应监督网络建设，重点加强对食品、饮品、水产品、旅游等市场管理，加大虚假广告的惩治力度，保证市民消费安全。强化对出国劳务、留学、人才、婚姻中介市场的规范和监管。开展“红盾护农”行动，对化肥、种子、农药全面实行目录管理制，切实保护农民利益。

　　八、大力发展各项社会事业，为构建和谐社会打下良好基础。坚持把教育摆在优先发展的地位，继续推进城区义务教育均衡发展，农村地区全部实行免费义务教育。大力发展职业教育，建成6个职业教育重点实习实训基地，扩大职业教育扶困助学覆盖面。进一步加强教育基础设施建设，完成大连一中、二十四中旧校舍改造和第十五中学异地新建工作。继续支持各类高校的建设与发展。

　　加快实施人才强市战略。健全人才工作政策，加强中高层次的经营型、创业型、技能型人才和国内外智力引进，重点强化农村科技、教育、卫生和实用型人才的引进和培养。继续办好海外学子创业周活动。加强高校毕业生就业指导工作，推进区市县毕业生就业指导服务机构建设，引导毕业生面向农村和基层就业。

　　加强公共卫生和医疗服务体系建设，整合优化医疗资源。加快疾控中心、传染病医院等重点项目建设。做好重大疾病预防控制工作，完善疫情监测预警系统建设。深入开展医院管理年活动，纠正医疗行业不正之风，重点在解决看病难、看病贵问题上取得成效。加强人口发展战略研究，完善人口和计划生育工作机制，继续稳定低生育水平。开展健康城市建设工作，提升城乡爱国卫生工作整体水平。

　　加快发展新闻出版、广播影视、文化艺术等事业。完成市区有线电视数字化整体转换40万户，开播移动数字电视，加快村村通有线电视的建设。深入开展全民健身活动，启动市体育中心规划建设工作。全面落实大连市妇女儿童发展规划，保障妇女儿童合法权益。深入做好双拥优抚安置、民族、宗教、侨务、对台、人防、民兵和预备役、残疾人及老龄事业等工作。

　　九、加强精神文明和民主法制建设，巩固和提升文明城市建设成果。实施《大连市创建全国文明城市工作规划（2006－2008年）》，进一步巩固文明城市创建成果，开展“我与文明城市同行”系列活动，继续加强“诚信大连”建设。积极推进道德、科教、文体、卫生、法律“五进社区”工作。加强社会公德、职业道德和家庭美德建设。进一步完善社区功能，完成市社区信息服务中心改造。

　　各级政府要自觉接受人大的法律监督、工作监督和政协的民主监督，坚持重大事项向人大常委会报告和与政协协商制度，畅通沟通联系渠道，充分听取各界的意见和建议，认真办理人大和政协的建议、提案。加强政府与工会联系，积极支持工会履行职能。认真贯彻《全面推进依法行政实施纲要》，推动行政机关依法行政，完善行政执法责任制，努力建设法治政府。完成第九批行政审批项目精简和全市年检制度改革工作。做好“五五”普法的规划和启动工作，深入开展法制宣传教育。进一步拓宽社情民意反映渠道，扩大基层民主，全面推行政务公开，切实保障广大人民群众依法行使民主权利。正确处理改革、发展、稳定关系，特别要注意解决好深化改革中涉及群众切身利益问题，落实好《信访条例》，认真做好信访和人民调解工作，积极化解社会矛盾。进一步规范和拓展法律服务。加快推进社会治安防控体系建设，提高城市治安整体防控能力，依法严厉打击各种犯罪活动，维护全市社会稳定，争创社会治安综合治理先进城市。坚持安全发展原则，进一步健全三级安全监管体系。强化安全生产基层和基础工作，推广安全生产质量标准化建设，落实安全生产问责制，建立市级事故应急救援中心、危险化学品集中控制中心，努力保持良好的安全生产形势。

　　十、加快政府职能转变，切实提高政府社会管理和公共服务能力。以推进政务公开为主线，加大软环境建设力度，提升政府的服务效能，完善和健全政府决策机制，努力提高行政决策的科学化、民主化水平，落实决策跟踪反馈和责任追究制度，保证决策的正确和有效。要全面加强各级行政服务中心建设，进一步提升电子政务服务水平，扩大服务范围。加快公共事件应急体系建设，提高应对突发事件的能力。提高政务公开、厂务公开、村务公开的针对性、规范性和互动性，全面推进教育、卫生、交通、水电气和物业等行业公开办事制度。全面贯彻实施《公务员法》，进一步规范公务员管理，提高公务员行政能力。全市各级公务员必须严格按照法定权限和程序行使权力、履行职责，牢固树立立党为公、执政为民的思想，做到权为民所用，情为民所系，利为民所谋。要大力弘扬“热爱祖国、忠于人民，恪尽职守、廉洁奉公，求真务实、开拓创新，顾全大局、团结协作”的公务员精神，加强学习，提高素质，为老工业基地振兴和构建和谐社会再立新功。

　　各位代表，我们已经站到了新的历史发展起点上，形势催人奋进，任务光荣艰巨。让我们紧密地团结在以胡锦涛同志为总书记的党中央周围，在省委、省政府和市委的正确领导下，解放思想，与时俱进，开拓创新，真抓实干，为推进我市经济社会持续较快健康发展而努力奋斗。
